# Oral Cancer Screening: A Biosocial Analysis of Global Barriers—A Narrative Review of Who Screens, Who Gets Screened, and Why

**DOI:** 10.3390/cancers18091381

**Published:** 2026-04-27

**Authors:** Razan M. Baabdullah, Lillian Gordon, Jordan Gigliotti

**Affiliations:** 1 Department of Oral and Maxillofacial Surgery, Faculty of Dentistry, King Abdulaziz University, Jeddah 21589, Saudi Arabia; 2 Department of Global Health and Social Medicine, Harvard Medical School, Harvard University, Boston, MA 02115, USA; 3 Harvard School of Dental Medicine, Harvard University, Boston, MA 02115, USA; 4 Department of Oral and Maxillofacial Surgery, Faculty of Dental Medicine and Oral Health Sciences, McGill University, Montreal, QC H3G 1A4, Canada

**Keywords:** oral cancer, early detection of cancer, healthcare disparities, biosocial analysis, healthcare policy, social determinants of health, mass screening, health services accessibility

## Abstract

**Background**: Oral cavity cancer remains a major public health challenge because many cases are diagnosed at advanced stages, when treatment is more complex and outcomes are poorer. Although oral cancer screening involves a simple, non-invasive examination, its implementation remains inconsistent across settings. This review examines barriers to oral cancer screening through a biosocial lens, with attention being paid to clinical, social, and policy factors shaping access. **Materials and Methods**: This study was conducted as a structured narrative review of peer-reviewed literature and policy documents. Targeted searches of PubMed and Google Scholar were taken from database from 2000 to May 2024 using MeSH terms and free-text terms related to oral cancer screening, disparities, social determinants, access, guidelines, and policy. Eligible sources included English-language studies and guidance documents addressing oral cancer screening practices, barriers, disparities, or recommendations. Findings were synthesized thematically and interpreted using a biosocial framework informed by social medicine theory. **Results**: The review finds that oral cancer screening is limited by variation in guideline recommendations, uneven integration across healthcare settings, and persistent disparities in access among high-risk populations. Patients with the greatest burden of risk often face the greatest barriers to early detection because of structural disadvantage, fragmented service delivery, and weak policy support. The literature also suggests that narrow evidentiary standards and cost-effectiveness debates may limit the legitimacy and uptake of screening initiatives. **Conclusions**: This review does not propose a wholly new model of screening; rather, it offers a biosocial interpretation of existing evidence to explain why screening inequities persist. More equitable oral cancer screening will require not only clinical attention to high-risk populations but also reforms in guideline development, service organization, and health policy to better address structural barriers to access.

## Simple Summary

Oral cancer remains frequently diagnosed at advanced stages, leading to poorer survival and greater treatment burden. We must make critical clinical and economic considerations for who screens, who gets screened, and why. Although screening is simple, non-invasive, and valuable for early detection, participation across healthcare specialties remains low, particularly among populations with the greatest exposure to risk factors such as tobacco, alcohol, and betel quid use. This review examines oral cancer screening through a biosocial lens, arguing that disparities in access are shaped not only by individual behavior but also by healthcare structures, screening guidelines, and broader social determinants of health. The analysis highlights how cost-effectiveness-driven policy approaches, entry points of limited screening, provider bias, and unequal access to dental care may exclude those at highest risk. It argues that more equitable screening requires broader provider training, policy reform, and stronger evidence focused on implementation and access.

## 1. Background

The nomenclature and reporting of oral cancer in scientific literature presents a significant methodological challenge, as many studies combine both oral cavity cancer and oropharyngeal cancer into a single category despite their distinct epidemiological patterns, screening protocols, etiological factors, and survival outcomes. This paper specifically addresses oral cavity and lip cancer, while carefully excluding studies that combine these conditions with oropharyngeal cancer except where explicitly noted. This distinction is crucial, as the current ambiguity in terminology impedes accurate interpretation of research findings and clinical recommendations.

Oral cancer encompasses malignant neoplasms of the mouth and lip tissues, with squamous cell carcinoma representing 90% of diagnosed cases [1,2]. Lip and oral cavity cancer, both referred to here as “oral cancer,” pose a global problem. While oral cancer is a treatable disease, its propensity for late diagnosis and subsequent high morbidity and mortality rates remains a widespread global concern [3,4,5,6]. While oral cancer is a treatable disease, its propensity for late diagnosis and subsequent high morbidity and mortality rates remains a widespread global concern. Prevention remains central to reducing the burden of oral cancer, with a recent prevention guide emphasizing risk-factor reduction, diet modification, ultraviolet protective measures, improvement of oral hygiene, public education, regular dental checkups, and early detection through routine oral examination as key components of oral cancer control [7]. Among the most prevalent oral cancer sites, early-stage disease in the US shows five-year survival rates of 94% for lip, 82% for tongue, and 76% for floor of mouth malignancies when localized [8,9,10]. However, these rates decrease precipitously in late-stage cancers with regional and distant metastasis, plummeting to 32%, 40%, and 20%, respectively [8,9,10].

Emerging research is exploring the potential of artificial intelligence-driven image analysis for early oral cancer detection [11]. This technology could empower untrained individuals to utilize smartphones for preliminary lesion assessment, distinguishing between high and low-risk cases. Such a tool has the potential to expand cancer screening access in resource-limited settings. Although various other screening methods exist, including light-based systems, brush cytology, and self-examination, the conventional oral examination remains the standard screening protocol [1].

Performed under adequate incandescent lighting, smartphone-based lesion assessments and examinations combine thorough visual inspection with palpation [1]. The Lingen et al. review “Critical Evaluation of Diagnostic Aids for the Detection of Oral Cancer” demonstrated the efficacy of this visual examination method, reporting specificity ranges of 0.75–0.94 and sensitivity ranges of 0.60–0.97 [1]. Yet, screening is performed at low rates [9] given that patients present at late stages [4,5,6]. According to the Surveillance Epidemiology and End Results (SEER), only about a quarter (26.4%) of patients are diagnosed with localized disease [8]. The delayed presentation to a clinic or specialized cancer center results in a lower survival rate [8].

Therefore, the oral cancer problem presents two fundamental challenges in contemporary healthcare. First, screening participation remains inadequately low across populations, particularly among low-income patients [9], limiting opportunities for early detection of oral cancer. Oral potentially malignant disorders (OPMD) may be difficult to detect clinically and can be challenging to distinguish from benign oral lesions [1]. Second, when diagnosed in advanced stages, oral cancer exhibits devastating mortality and morbidity rates, with significantly diminished survival outcomes [9], along with higher economic burden compared to early stages [9,12].

Analysis of Medicaid Expansion under the Affordable Care Act (ACA) demonstrates higher rates of early-stage oral cancer detection in expansion states compared to non-expansion states [9,13,14]. These findings suggest that improved healthcare accessibility leads to earlier diagnosis through increased screening opportunities. The imperative for enhanced screening protocols therefore emerges as a critical public health priority.

Several authors in the oral cancer screening domain advocate focusing all efforts on screening an at-risk group, i.e., current smokers, alcohol users or betel quid (areca nut) chewers [15,16]. However, an important issue remains insufficiently addressed: whether these populations can actually access screening in practice. Although at-risk targeted screening is frequently recommended, less is known about how healthcare organization, provider roles, and screening guidelines may limit access for the very groups identified as highest risk. In particular, screening opportunities (entry points) may be restricted by narrow clinical entry points and by guidelines that limit which practitioners are expected or authorized to perform screening [17,18,19].

This paper employs a narrative review methodology, a format particularly suited to complex public health problems that cut across clinical, epidemiological, and social science evidence, and where a single study design cannot tell the full story [20]. This approach allows us to bring together quantitative evidence on screening disparities, guideline documents from major regulatory bodies, and social medicine frameworks within a single analytical qualitative lens.

A biosocial analysis examines how social and biological factors interrelate across the life course to shape patterns of disease, rather than treating health outcomes as solely biomedical or solely social [21]. Recent cancer disparities scholarship describes biosocial determinants as emerging at the intersection of lived social experience and individual biology, helping explain why inequities in cancer incidence, survival, and outcomes persist over time [21]. Applied to oral cancer screening, this perspective is useful because it directs attention not only to individual risk behaviors, but also to how structural disadvantage, healthcare access, and institutional arrangements become reflected in unequal opportunities for early detection and diagnosis [21].

The aim of this paper is to provide a biosocial analysis of the barriers to oral cancer screening globally, addressing three focused questions: (1) what the current screening practices and entry points are, including who is screened and which healthcare providers conduct screening; (2) what guidelines and protocols are recommended by major international bodies; and (3) what barriers prevent those at highest risk from accessing screening services. In doing so, the paper goes beyond describing the evidence to interrogate the assumptions and power structures embedded in how that evidence has been produced and institutionalized—and to examine how clinical recommendations, healthcare systems, and structural disadvantage interact to shape who gets screened and who does not. The analysis then offers practical recommendations for improving existing screening services while addressing systemic barriers to access to encourage more equitable and effective oral cancer screening programs across different healthcare settings.

### Incidence, Mortality Rate, and Global Burden of Oral Cancer

Oral cancer was reported as the 16th most common cancer in the world in 2022, in both incidence and mortality [22]. It is 2.5 times more common in men than in women worldwide; however, the incidence of oral cancer among women is trending up in countries where tobacco smoking is on the rise [23]. The World Health Organization (WHO) Global Oral Health Status Report indicates that the incidence of oral cancer directly follows the trends in the incidence of tobacco smoking or chewing and use of betel quid (areca nut) geographically [24].

Both high- and middle-income countries bore almost the predominant burden of oral cancer with regard to the number of cases in 2020 according to the WHO’s Global Oral Health Status Report in 2023 [23].

From 1990 to 2017, the global burden of oral cancer exhibited an upward trend [24]. The Global Burden of Diseases (GBD) 2019 Lip, Oral, and Pharyngeal Cancer Collaborators [25] assessed the global burden of oral cancer spanning from 1990 to 2019, finding that both low and low–middle regions suffered from the highest age-standardized mortality rate. The new cases of oral cancer in 2019 were 370,000, and deaths were estimated to be 199,000, resulting in DALYs of 5.45 million [25]. India alone accounts for 136,000 new cases, which exacerbates the global burden of non-communicable diseases (NCDs) [23]. Recent analysis of the Indian subcontinent reports that the region accounts for a disproportionately high share of global deaths and disability-adjusted life years (DALYs) from lip and oral cavity cancers, highlighting the need for urgent policy attention to prevention, early diagnosis, and management in high-burden settings [26].

## 2. Materials and Methods

### 2.1. Review Design

This study offers a structured narrative review guided by a biosocial analytical framework. The purpose of the review is not to produce an exhaustive systematic review or meta-analysis, but to synthesize clinical, public health, and policy literature relevant to oral cancer screening and to examine barriers to implementation beyond purely biomedical explanations. A narrative review design was selected because the research questions are interpretive and multidisciplinary, spanning epidemiology, screening practice, health policy, disparities, and social medicine.

The review is structured around five questions:

1. What are the key risk factors for oral cancer that justify the need for screening?
2. What are the current screening practices and entry points for oral cancer detection?
3. What screening guidelines and protocols are recommended by major national and international bodies?
4. What biosocial barriers prevent those at highest risk from accessing screening services?
5. How might screening programs be reformed to provide more equitable and effective detection?

### 2.2. Search Strategy

A targeted literature search conducted in PubMed and Google Scholar identified relevant peer-reviewed literature, supplemented by a search for policy statements and guideline documents from major regulatory and professional bodies. Searches covered publications inputted into the database from 2000 to May 2024.

In PubMed, both Medical Subject Headings (MeSH) and free-text keywords improved search sensitivity and specificity. MeSH terms included: “Mouth Neoplasms,” “Mass Screening,” “Early Detection of Cancer,” “Health Services Accessibility,” and “Healthcare Disparities.” These are combined with free-text terms including: oral cancer screening, oral cancer screening guidelines, oral cancer disparities, social determinants of oral cancer, oral cancer screening access, and oral cancer policy. Additional reference list searching of key articles identified relevant sources not captured in the initial search.

Guideline and policy documents were also identified from recognized organizations, including the World Health Organization (WHO), International Agency for Research on Cancer (IARC), U.S. Preventive Services Task Force (USPSTF), American Dental Association (ADA), and British Dental Association (BDA).

### 2.3. Eligibility Criteria

Sources were included if they:

(1) Were published in English;
(2) Reported on oral cancer screening practices, access, barriers, disparities, or screening-related recommendations;
(3) Included original studies, systematic reviews, narrative reviews, or policy/guideline documents relevant to oral cavity cancer screening.

Sources were excluded if they:

(1) Focused exclusively on oropharyngeal cancer without separately reporting oral cavity findings or clearly distinguishing the two disease entities;
(2) Were case reports, commentaries, or editorials without substantive primary or policy-relevant content;
(3) Were not directly relevant to the review questions.

Because this is a narrative review, formal risk-of-bias scoring and quantitative pooling were not undertaken.

### 2.4. Study Selection and Data Handling

Retrieved articles and documents were screened for relevance based on title, abstract, and full-text review where needed. Data extraction was interpretive rather than statistical and focused on the following elements: screening setting and provider, target population, barriers to screening, disparities in access or outcomes, recommendations from regulatory bodies, and implications for health policy.

### 2.5. Narrative Synthesis

Findings were synthesized narratively and grouped thematically according to the five review questions. The literature was organized into four broad domains:

(1) Screening practices and entry points;
(2) Disparities in access and uptake;
(3) Variability in guidelines and recommendations; and
(4) Structural and policy determinants of screening implementation.

These themes were interpreted through a biosocial lens to examine how biological risk, healthcare systems, and sociopolitical context interact to shape who is screened, who is missed, and why.

### 2.6. Conceptual Framework

The analysis was guided by a biosocial framework drawing on Arthur Kleinman’s social theories for global health [27], described in Section 3, to examine how biological and social factors interact to influence oral cancer screening outcomes.

Two concepts informed our interpretation of the literature:

(1) Social suffering, referring to the ways in which socioeconomic and political forces generate conditions that may increase disease burden [27]; and
(2) Social institutions and healthcare bureaucracies: although structured around guidelines and protocols intended to promote positive change, social institutions and healthcare bureaucracies can give rise to unintended outcomes, spanning both beneficial and adverse consequences [27].

This framework links biological risk with structural determinants of oral cancer, accommodates the interdisciplinary nature of screening disparities, and provides a consistent interpretive lens for examining guideline formation, healthcare access, patient agency, and the political economy of screening. In this review, these concepts were interpreted in relation to observable indicators reported in the literature, including screening uptake, stage at diagnosis, treatment patterns, and survival differences.

## 3. Results and Discussion

### 3.1. Oral Cancer Risk Factors and Clinical Imperative

The International Agency for Research on Cancer (IARC) indicated that the most significant modifiable risk factors for oral cancer are smoked and smokeless tobacco, areca nut use in South and East Asia, and high alcohol consumption [28]. Approximately 90% of oral cancers globally are estimated to be caused by smoking, alcohol consumption, and poor nutrition [29], although the role of nutrition may be preventive [30]. Tobacco and alcohol consumption in combination has a synergistic effect, increasing the risk of oral cancer by thirty times [31]. Lee et al., in their “Risk Prediction Models for Head and Neck Cancer in the U.S. Population from the INHANCE Consortium,” reported that the 20-year absolute risks among smokers for oral cancer increased from 2.73% to 4.60% for men and from 2.61% to 3.54% for women [32].

Smoking cessation is correlated with a reduced risk of oral cancer. The longer one refrains from smoking, the lower one’s risk of developing oral cancer [28]. In their meta-analysis, Marron et al. found that the incidence of oral cancer decreased by 35% among former smokers who abstained from smoking for one to four years compared to current smokers [33]. The incidence of oral cancer decreases to the level of a never-smoker if cessation is continued for 20 or more years [28,33,34,35]. The IARC also established that cessation of smokeless tobacco and areca nut products decreases the incidence of oral cancer and premalignant lesions [28,30].

HPV infection incidence has increased among young white men, contributing to a global increase in HPV-related oropharyngeal cancer, rather than oral cancer alone [23]. Although HPV-related oropharyngeal cancer represents a distinct etiological pathway requiring different screening and preventive approaches, several studies indicate that HPV vaccination for both males and females may be a primary prevention method for both oral cavity and oropharyngeal cancer and is a strategy followed in some countries [23,27]. However, due to the relatively recent advent of HPV vaccination, there is insufficient empirical data to establish its effectiveness in preventing oral cavity and oropharyngeal cancer [27].

Other risk factors for oral cancer include genetics, age, and environmental factors [28,36]. Warnakulasuriya and Greenspan [37] also link oral cancer to deprivation and low socioeconomic status (SES) [37]. In fact, the IARC Handbooks of Cancer Prevention underscores the critical influence of SES, as well as sociocultural determinants, in not only initiating behaviors like smoking, but also perpetuating their continued use [30].

### 3.2. Current Oral Cancer Screening Practices

#### 3.2.1. Understanding Low Oral Cancer Screening Rates:

Several studies have identified multiple factors contributing to the low rate of oral cancer screening, including lack of awareness among the public on modifiable risk factors [28], disparities in access to care [17], biases of healthcare professionals in screening for oral cancer [38], knowledge, attitude, and practice (KAP) of general dentists or other healthcare providers who may be unfamiliar with the signs and symptoms of the disease and disease presentation, lack of well-defined referral pathways to a specialist when needed, or to the simple fact that general dentists do not routinely screen for oral cancer [39,40,41].

Additionally, socioeconomic substrates and ethnicities inadvertently serve as proxies in determining who gets screened for oral cancer [17]. These factors also erroneously seem to determine who is likely to present at later stages and who has a higher predisposition for lifestyle-related risk factors [17,19,22]. For example, 72.2% of patients who are diagnosed with oral cancer present in advanced Stages III and IV in the U.S. [7]. Globally, oral cancer presents a grave challenge, with nearly half of all oral cancer cases being diagnosed at an advanced stage (Stages III and IV) [4,5,6].

These concerning statistics reflect a complex interplay of factors as indicated above: while oral lesions are relatively common, distinguishing potentially malignant cases remains challenging since conventional examinations cannot reliably identify progressive versus benign lesions [1], and some precancerous changes may be present even in clinically normal-appearing tissue [1], further compounded by systemic gaps in routine screening practices [39,40,41].

**Figure 1:**
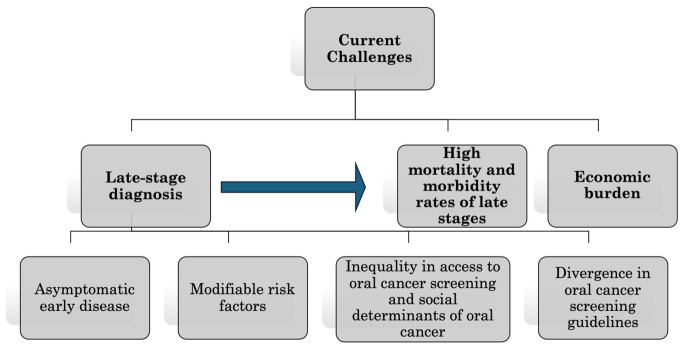
Summary of the Current Oral Cancer-Related Challenges: Oral cancer presents dual healthcare challenges: late-stage diagnosis combined with poor survival outcomes and high economic burden.

Considering rising cancer rates, Gales et al. [42] advocate for prioritizing universal oral cancer screening programs in all low- and middle-income, a critical intervention that forms the focus of this paper (Figure 1).

#### 3.2.2. Who Has Access to Screening?

Understanding oral cancer risk factors is critical for two main reasons. First, the modifiable nature of many of these factors underscores the potential for preventive interventions to be efficacious. Second, individuals characterized by lower SES often manifest a heightened prevalence of lifestyle-related and modifiable risk factors, such as tobacco and alcohol use [17,18,38,43]. Consequently, they stand to benefit significantly from oral cancer screening initiatives more than others. Oral cancer screening provides opportunities to promote appropriate lifestyle interventions. However, the confinement of oral cancer screening to opportunistic avenues, such as those provided within dental care settings, imposes access barriers, particularly for individuals already facing impediments to healthcare services.

Broadening access to oral cancer screening has the potential to ensure equitable healthcare access especially considering the potential advantages of screening as indicated by Warnakulasuriya and Kerr [36]: (1) the possibility of detecting oral cancer at earlier stages; (2) potential reduction in morbidity and mortality associated with oral cancer; and (3) the opportunity to identify OPMD [36]. In contrast to invasive approaches employed in other cancer screening methodologies, the oral cancer screening examination is composed of both palpation and visual inspection of the lip and oral cavity under illumination, and a neck exam to identify any lymph node suspicious for metastasis [28,36]. The goal of screening is not to establish a definitive or even a preliminary diagnosis but rather to identify any abnormal oral mucosal findings and make the necessary referral to a head and neck specialist in a timely fashion [36].

#### 3.2.3. Entry Points: Who Screens for Oral Cancer?

After reviewing the body of evidence on oral cancer screening, the IARC indicates that screening individuals with a higher risk for oral cancer could decrease the incidence of oral cancer mortality [28]. IARC proposes that screening could be performed by trained primary healthcare providers (PHCPs) in low-resource settings or by dentists, for example, as part of “opportunistic screening” in high-resource areas [28]. In considering a nation characterized by a high socio-demographic index, such as the U.S., and within it, a state like Massachusetts, deemed affluent in resources, it is striking to note that one in four patients in Massachusetts do not have dental insurance, according to the Massachusetts Health Policy Commission [44]. This indicates the paucity of dental care services accessible to marginalized communities. Thus, training healthcare providers beyond just dentists to screen for oral cancer will help create more entry points for screening, regardless of a country’s economic level. Warnakulasuriya and Kerr [36] report that when access to dental care or trained physicians is limited in middle- and low-income countries, training PHCPs to screen at-risk populations for oral cancer has been shown to be feasible and cost-efficient [36].

While screening high-risk groups for oral cancer in opportunistic settings can be cost-efficient, it is not beneficial for patients with no access to dental care or those who do not visit their PHCPs regularly [36]. Warnakulasuriya and Kerr recommend the following measures to increase opportunistic screening for high-risk individuals: (1) provision of training for oral cancer screening; (2) improvement of the undergraduate teaching for medical, dental, nursing, and other healthcare providers; (3) development of digital learning tools to train those who have the desire to screen; (4) establishment of “national practice-based networks” to collect data, conduct research, and assess current practice habits; and (5) development of a risk-evaluation model to help assess risk profiles [36].

However, if the national bodies fail to recommend training for PHCPs to screen for oral cancer, it could result in a lack of motivation among PHCPs or other specialties to perform the screening. This is due to the ways in which agendas are set and recommendations are made by regulatory bodies, which can influence the attitudes and practices of healthcare providers.

#### 3.2.4. Review of Major Oral Cancer Screening Protocols and Recommendations from National and International Bodies

The UK National Screening Committee [28] and the U.S. Preventive Services Task Force (USPSTF) [45] do not recommend screening asymptomatic individuals for oral cancer. It is salient to note that the recommendation by the USPSTF was directed to providers of primary medical care rather than to dentists or otolaryngologists. Instead, dentists and otolaryngologists should perform a comprehensive head and neck exam to screen for oral cancer [36]. Similarly, the American Cancer Society [46] made no recommendations for routine screening for oral cancer for asymptomatic patients. However, the American Dental Association (ADA) [47] and the British Dental Association (BDA) [48] recommend that all dentists should screen all adult patients for oral cancer regularly, during every course of dental treatment, including during emergency visits [36]. This also illustrates an unintended consequence of purposive action. Although screening recommendations are designed to reflect evidentiary standards and avoid low-value interventions, they may produce measurable downstream effects by narrowing where screening is expected to occur. In practice, this can widen the gap between patients who encounter screening in dental settings and those who rely mainly on primary care settings, thereby reproducing unequal screening opportunity across the healthcare system.

The diversions in recommendations give rise to several challenges. First, they amplify the disparity in access to oral cancer screening services. Patients with access to dental care should be screened per the ADA’s and BDA’s recommendations [47,48]. In contrast, those without access to dental care may not receive routine screening until the disease has progressed to an advanced stage [17,18]. This perpetuates disparities and does not advocate for equality in access to healthcare services. More inclusive screening programs then become a necessity as an essential component of universal health care.

Second, Warnakulasuriya and Kerr [36] recommend increasing access to oral cancer screening by training more healthcare providers. However, healthcare providers will have to follow the guidelines set by their regulatory body. For example, training more PHCPs to do oral cancer screening may be hindered by resistance because the USPSTF [45] fails to mention routine screening for oral cancer in primary care. Hence, it does not matter how many training programs are out there if regulatory bodies do not recommend them, as they hold a position of legitimacy. Legitimation explains how policies and practice guidelines are institutionalized [49]. PHCPs then must navigate the interplay between what has been legitimized and institutionalized and what evidence-based practice warrants.

Third, recommendations are expected to align with evidence-based medical practices. However, cost efficiency [28] seems to be a vital metric in making recommendations, which may limit access to care, especially for those with already limited access. The implication of centering recommendations on cost efficiency will be detailed later in this paper.

A critical observation—and one that is central to this paper’s epistemological argument rather than its evidentiary basis—is that many of the major guideline bodies’ recommendations are based on a single randomized controlled trial (RCT) by Sankaranarayanan et al. in one city, Kerala, India [15]. This study is the only RCT on oral cancer screening in the literature to date that studied oral cancer mortality as the endpoint. Despite some significant limitations in the design, it has become a pivotal paper on which many researchers have built their work. In spite of the generalizability bias, recommendations continued to emerge from it to uphold their evidence-based practice. Because of their well-regarded position on the hierarchy of evidence, as they are at the highest level of knowledge production [50], RCTs are viewed as an evidence-based science that guides clinical decisions and influences guideline development. Perhaps it is essential to examine and scrutinize how evidence-based medicine is construed.

#### 3.2.5. Biosocial Analysis of the Problem

A biosocial analysis unpacks how biological and social factors interact to shape health outcomes and disparities in disease patterns [21]. Through theoretical frameworks like Arthur Kleinman’s social theories [27], particularly his concepts of social suffering and unintended consequences of purposive action, we can understand how socioeconomic and political forces can create environments that may increase disease burden. This framework illuminates how oral cancer becomes “a disease of the poor and dispossessed” [51], shaped by structural violence and institutional practices. The analysis also considers the “Hegemonic Medical Model” [52], which prioritizes biological factors over social determinants in healthcare policy formation. This comprehensive approach reveals how social determinants of health, access to treatment, healthcare provider biases, and political economy collectively influence oral cancer outcomes, moving beyond simplified narratives of patient behavior to examine the broader structural forces that shape disease patterns and healthcare delivery. The analysis below reinforces two critical challenges in oral cancer that were previously discussed: patients are typically diagnosed at advanced stages, leading to both high mortality rates and substantial financial burden on healthcare systems.

### 3.3. Oral Cancer Disparities and Social Medicine Practices

The National Cancer Institute [53] refers to cancer disparities as “differences in cancer measures,” including incidence, prevalence, mortality rate (cancer-specific), morbidity rate, survival rate, screening rates, stage at diagnosis, and the financial burden of cancer. Oral cancer disparities persist not only in incidence, prevalence, mortality rate, and burden of the disease but also in the attainment of healthcare quality. Sofi-Mahmudi et al. [24], who evaluated the global burden of lip and oral cancer and its corresponding quality of care from 1990 to 2017, report that African nations (including Somalia, Eritrea, Central African Republic, Lesotho among the lowest) exhibit the lowest score for the quality of care, contrasting with the highest quality of care observed in Western European countries, Australia, and North America. These countries also exhibit the lowest DALYs, while those with the low–middle socio-demographic index countries experience the highest DALYs. Despite advancements in oral cancer treatment, equitable access to the highest level of care varies dramatically among countries.

Arthur Kleinman discusses four social theories that help guide the practice of global health and social medicine: unintended consequences of purposive action, social construction of reality, social suffering, and biopower [27]. For the purposes of this paper, we will focus on two of these theories. First, social suffering holds that socioeconomic and sociopolitical forces can create environments that increase the incidence of the disease [27]. In the case of oral cancer, social suffering may exacerbate oral cancer progression, as evidenced by the predominance of late-stage disease presentation [17,19]. As Johnson et al. [51] put it, “Oral cancer is a disease of the poor and dispossessed.” It is a product of social suffering and structural violence [27]. Second, social institutions and healthcare bureaucracies, despite aiming for positive change through guidelines and protocols, can produce unintended consequences, both beneficial and harmful. Like Robert Merton theorized, all social interventions spawn unintended consequences, some predictable and preventable, others not [27]. This demands constant evaluation of our healthcare systems’ actual impacts, ready to modify or discard policies when their unplanned effects undermine their intended benefits.

Furthermore, the “Hegemonic Medical Model” [52], which pertains to the ways in which healthcare policies are shaped, packaged, and propagated, describes the epistemological structure of policymaking where biology is primary, and the social system is secondary. Policymakers play a vital role in promoting health equity and social justice by addressing social determinants of health in their recommendations. The following studies show that the social determinants of oral cancer can no longer be neglected.

### 3.4. Examining the Intersection Between Social Conditions, Healthcare Access, and Oral Cancer Outcomes

Agarwal et al. [17] were the first to study the relationship between oral cancer and social determinants of health and Medicaid status in the U.S. Their retrospective study used the SEER database to collect demographic and cancer-related data. They evaluated SES as part of the social determinants of health (SDH), and they used health insurance status as a proxy for SES [17]. Their findings revealed that Medicaid patients had a poor prognosis, presenting at later stages with advanced tumor size and regional or distant metastases. They were also unlikely to get surgical or definitive treatment. Instead, they were mostly referred for chemotherapy or radiation therapy compared to insured patients [17].

It is important to note that non-surgical treatment is the most common treatment among the Medicaid group, not only because of the advanced disease that makes cancer not amenable to resection, which may be a problem of access, but also because of the severe medical comorbidities that make this group less likely to be a candidate for surgical treatment [17]. Social conditions thus peel themselves layer by layer in this study. The severe medical comorbidities inherent in the Medicaid group’s overall health should not be overlooked, as they express the deleterious effects of inequality and other social problems. It is as though Medicaid is a poor prognostic indicator and emblematic of a grave complication. The unadjusted five-year survival rate was worse for Medicaid patients (40.7%) than for insured patients (67.7%) [17]. This disparity can be understood as a measurable expression of social suffering within the biosocial framework. Here, social disadvantage is not only described abstractly, but also appears in quantifiable differences in prognosis and survival. The markedly lower five-year survival among Medicaid patients suggests that structural vulnerability becomes embodied in worse cancer outcomes.

A Brazilian epidemiological study by Ramos et al. [19] studied the incidence and mortality rate related to oral cancer in Brazil from 2000 to 2019. They found that black patients had education levels lower than those of other racial groups. They were also the ones to suffer increased exposure to risk factors such as smoking and alcohol, were diagnosed at advanced stages, and had a higher mortality rate compared to patients from other racial groups [19]. One significant factor contributing to late diagnosis was the lack of access to healthcare [19,54]. This raises a crucial question of access. What factors contribute to these low-income groups being diagnosed at a more advanced stage? Analyzing sociopolitical and socioeconomic forces may provide some answers.

### 3.5. Victim Blaming: Patients’ Lack of Knowledge, Agency, and Risk Factors

Notwithstanding the improvements in oral cancer diagnosis and treatment over the years, the race gap remains unclosed [8]. The Centers for Disease Control and Prevention (CDC) indicate that the five-year survival rate for black men is 41% compared to white men, which is 62% [55]. Patients’ lack of knowledge has been reported as a cause of or explanation for the decrease in the five-year survival rate among different races [56].

While a dearth of knowledge may contribute to the issue, it merely grazes the surface of the underlying malaise. Furthermore, the real issue stems from a profound and systemic predicament entrenched in historical contexts and exacerbated by austerity measures and adverse social and working conditions. Inequalities, lack of access to healthcare, or biases within the healthcare framework, for example, serve as the true genesis of the problem.

Ascribing the problem to a lack of knowledge will not help solve it, despite the keen efforts to do so. Blaming the patient for purported non-compliant or attitudinal shortcomings fails to address the crux of the matter, as it disregards the impact of sociohistorical factors that shaped their attitude or behaviors, including habits such as smoking. It thus becomes imperative to contemplate the extent of agency patients possess within the framework of their sociohistorical context.

External forces may affect a person’s agency. For instance, while it is known that habits and behaviors adversely impact health and cause oral cancer, powerful forces can manipulate behaviors, like the marketing of cigarettes in African American neighborhoods [57]. Mukherjee writes in her book, Understanding and Practicing Social Medicine [57]:

Despite the constrained agency of impoverished and oppressed people, public health programs continue to focus significant attention on what are called information, education, and communication (IEC) campaigns that seek to modify individual behavior and neglect intervention to mitigate adverse social forces [57].

These inherent problems in the system require more than just one healthcare provider to overcome. For example, advising patients to stop smoking, as it is a risk factor for oral cancer [51], when they lack a support system to assist them in quitting, will not alleviate the suffering or reduce the burden of the disease. Similarly, recommending a healthy diet, which is also a risk factor for oral cancer [51], when they live in food deserts and lack access to quality food, will not treat the root cause of the problem.

### 3.6. Who Gets Screened? Healthcare Providers’ Biases

Another problem with assigning causality to patients’ lack of knowledge is the potential influence of healthcare professionals’ biases on the declining survival rates. As evidenced by Gupta et al.’s study [38], even upon accessing dental care, individuals from minority racial and ethnic groups encountered a reduced likelihood of oral cancer screening by their dentists, solely due to their race or lower SES. Their research highlights the importance of performing oral cancer screening for all patients, irrespective of their race, ethnicity, substance use, and socioeconomic status [38]. Within the biosocial framework, this pattern may be interpreted as a quantifiable expression of biopower. Even when patients reach a clinical setting where opportunistic screening could occur, institutional practices and provider decision-making still shape whose risk becomes actionable. In this case, race, insurance status, and socioeconomic position appear to influence who is actually screened, despite nominal access to care.

Gupta et al. assessed who gets screened for oral cancer across the U.S. They used data spanning from 2011 to 2016 from the National Health and Nutrition Examination Survey (NHANES). Their analysis disclosed that patients of minority racial or ethnic groups, less educated patients, low-income patients, uninsured patients, and Medicaid-insured patients were less likely to receive oral cancer screening during their dental visit compared to white, non-Hispanic groups and privately insured patients. Despite having access to dental care, these patients were devoid of the right to receive opportunistic screening for oral cancer due to ethnic and socioeconomic disparities in the healthcare system [38].

These disparities are compounded by the increased prevalence of lifestyle-related modifiable risk factors, such as tobacco and alcohol use, among these populations [18,38,43]. Therefore, they merit preferential (fast-track) examination for oral cancer screening more than others. Paul Farmer called it the “preferential option for the poor” [58].

Akinkugbe et al. [18] conducted a study to evaluate whether patients with lifestyle-related and modifiable risk factors would receive oral cancer screening exams using data from the NHANES between 2013 and 2016 across the U.S. Unfortunately, current smokers were less likely to receive oral cancer screening during dental visits and were even less likely to report seeing a dentist in the previous year. Akinkugbe et al. [18] posit and rationalize that high-risk individuals are less likely to refer themselves to specialists. If they do seek healthcare, it is more likely to be a PHCP rather than a dentist. This then raises the question of what societal barriers impede their access [18]. Another critical note is that training PHCPs is a much-needed intervention. Akinkugbe et al. further elaborate that smokers are disproportionately burdened by lower education, SES, and non-private insurance [18]. This corroborates the findings of Gupta et al. [38] and Ramos et al. [19]

Global oral cancer screening programs seem to prioritize cost-effectiveness over understanding social determinants, historical context, risk behaviors, and healthcare accessibility among vulnerable populations.

### 3.7. The Importance of Political Economy and Cost-Effectiveness in Agenda Setting

This review does not undertake a formal health economic evaluation. Instead, it considers how cost-effectiveness reasoning is used within screening policy debates and how such reasoning may influence the legitimacy and implementation of screening programs.

A common theme in the literature, evident in the IARC report [28], is that screening for oral cancer can detect the disease early but is only cost-efficient when performed for at-risk populations. Cost-effectiveness is a legitimate and necessary consideration in healthcare resource allocation, particularly in low- and middle-income settings where competing health priorities are acute.

However, when global health stakeholders prioritize cost-efficient interventions, they run the risk of missing the root causes of maladies and neglecting understudied and marginalized populations [59]. When applied as the primary filter for guideline recommendations, cost-effectiveness reasoning can obscure the broader economic case for screening.

In fact, the focus on cost-efficiency is paradoxical, as late-stage oral cancer diagnoses in the United States not only diminish survival rates but also incur substantially higher treatment costs, more than $10,000 higher than early stages [9,12].

Pierson and Verguet [59] underscore the importance of considering uncertainties and prior beliefs in decision-making, suggesting that funding more uncertain interventions could yield valuable and impactful solutions to global health and rampant problems. They argue that cost-efficient interventions may inadvertently overlook more consequential opportunities [59].

In that regard, we can closely examine Paul Farmer’s argument [60]. He contends that delivering high-quality care is not only a matter of social justice but can also lead to innovative fixes as opposed to adopting the “cost-effective” mentality [60]:

Treating poor Peruvians who suffer from multidrug-resistant tuberculosis according to the highest standard of care, rather than according to whatever happens to be deemed “cost-effective,” is not only social justice work but also, ironically enough, innovative [60].

Changing oral cancer screening guidelines is inherently entwined with the fabric of politics. Politics cannot be separated from medicine, as Rudolf Virchow stated [61]: “Medicine is a social science, and politics is nothing else but medicine on a large scale.” The crux of the matter lies not in cost-efficiency or return on investment, but rather in the equitable distribution of benefits within what is ostensibly labeled as an efficient system. The infiltration of cost-effectiveness into medical practices yields detrimental repercussions across multiple domains, effectively commodifying care, as poignantly observed by Arthur Kleinman [62] in The Soul of Care. Central to the discourse is the inquiry: are the recommendations of major regulatory bodies inherently driven by cost efficiency, or is this merely an incidental outcome? What ramifications ensue from recommendations steeped in cost-effectiveness? Moreover, by eschewing a cost-centric approach, how might recommendations evolve to prioritize factors beyond statistical significance and *p*-values, instead considering social determinants of oral cancer prevention and treatment?

### 3.8. Recommendations and Future Directions

The intractable nature of oral cancer disparities represents a fundamental challenge to contemporary healthcare systems, transcending mere screening inadequacies to encompass profound systemic inequities. The predominance of advanced-stage presentations emerges from an intricate confluence of factors: the insidious progression of early-stage pathology, heterogeneous healthcare access patterns across provider modalities, and deeply entrenched social determinants of health. Furthermore, the contentious discourse surrounding the expansion of oral cancer screening beyond traditional dental practitioners (professional territoriality) illuminates critical tensions between professional autonomy and public health imperatives, a paradigmatic example of how institutional inertia can impede crucial public health initiatives. Isolated screening programs may be inadequate in addressing the multifaceted nature of oral cancer disparities.

Historical precedent provides invaluable insights into both the promise and limitations of previous interventions. The American Dental Association’s (ADA) [63] two-pronged campaign launched in 2001, and the statewide model in Maryland [64] to prevent oral cancer and promote its early detection, serve as compelling case studies in the critical importance of sustainable funding mechanisms and systematic implementation frameworks.

**Figure 2:**
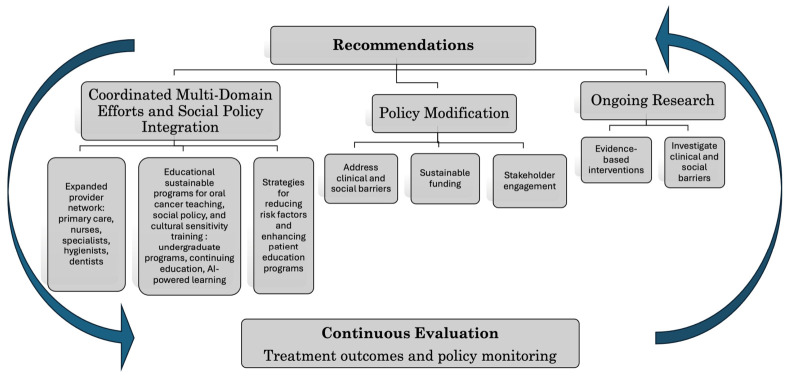
Three Pillar Recommendation Framework: The three-pillar framework includes coordinated multi-domain efforts and social policy integration, policy modification, and ongoing research. The arrows represent the iterative process of surveillance and underscores continuous evaluation.

We propose a tripartite framework for intervention, encompassing: (1) multi-domain initiatives and social policy integration, (2) systematic policy modification, and (3) rigorous empirical investigation (Figure 2). These recommendations are intended as an implementation-oriented framework, with each pillar linked to specific actors, actions, and measurable indicators.

The first pillar necessitates a comprehensive reconceptualization of provider networks in early detection. This expansion must transcend traditional disciplinary boundaries to incorporate primary care physicians, nurses, family medicine practitioners, dental hygienists, and otolaryngologists, while establishing robust referral infrastructures that facilitate seamless care coordination. The implementation of sophisticated training paradigms across the medical education continuum becomes paramount, leveraging cutting-edge technological innovations, including artificial intelligence-enhanced learning platforms and immersive virtual reality simulations. In practice, this should include standardized opportunistic oral examination in dental clinics, primary care, community health centers, tobacco cessation services, and otolaryngologist pathways serving high-risk populations. The implementation of sophisticated training paradigms across the medical education continuum becomes paramount, leveraging technological innovations, including artificial intelligence-enhanced learning platforms and immersive virtual reality simulations. These educational initiatives must extend beyond clinical competencies to address implicit bias recognition, cultural sensitivity, and social determinants of health, building upon successful frameworks. Progress in this pillar should be assessed through measurable indicators such as the proportion of providers trained, the proportion of routine visits in which screening is documented, referral completion rates for suspicious lesions, and stage at diagnosis among screened populations. Additionally, public health initiatives should integrate screening with tobacco cessation and HPV vaccination campaigns, while partnering with local and community organizations to reduce risk factors and enhance patient education.

The second pillar, policy modification, calls for a systematic restructuring of healthcare delivery systems to ensure equitable access to high-quality care, regardless of socioeconomic or insurance status. This involves addressing clinical and social barriers to early diagnosis while fostering meaningful engagement with stakeholders, including healthcare providers, insurers, policymakers, and community leaders, to secure sustained funding and political support. In operational terms, policy reform should specify which providers are expected to perform opportunistic screening, under what circumstances referral is indicated, and how screening and follow-up are funded within health systems.

Policy advocacy must prioritize evidence-based screening protocols, comprehensive insurance reform, strategic workforce development, and the seamless integration of oral health into primary care frameworks. The success of this pillar can be evaluated through the adoption of revised guideline language, the introduction of reimbursement mechanisms for screening and referral, the number of health systems integrating oral cancer screening into routine care pathways, and reductions in screening disparities by insurance status, income, race, or geography.

The third pillar emphasizes the critical importance of ongoing empirical investigation into the barriers to early diagnosis and the efficacy of interventions. This includes developing evidence-based strategies that address the full spectrum of treatment outcomes and morbidities across various stages of the disease. While immediate action is essential to counter the current reality of predominantly late-stage diagnoses, it must be balanced with methodologically rigorous prospective studies that systematically evaluate screening programs and intervention outcomes. Such evaluation should move beyond overall screening volume to examine whether screening reaches populations at highest risk and whether it improves downstream outcomes, including referral completion, time to biopsy or diagnosis, stage at presentation, treatment initiation, and, where feasible, survival. This research agenda recognizes the complexity of healthcare systems and the potential for unintended consequences, emphasizing the need for flexibility to adapt or abandon interventions that prove ineffective or harmful, regardless of their theoretical merit. A practical pathway would therefore begin with pilot implementation in high-yield settings, followed by standardized referral protocols, reimbursement support, and prospective equity-focused evaluation before wider scale-up.

The implementation of this comprehensive framework requires unprecedented coordination across multiple domains of healthcare delivery and social policy. Success demands not only technical expertise but also political will and sustained resource allocation. The human cost of continued inaction, measured in preventable morbidity and mortality, renders the status quo untenable. Therefore, we assert that immediate, coordinated action across these three pillars represents not merely an opportunity for healthcare system improvement, but a moral imperative for the medical community.

### 3.9. Limitations of the Review Method

As a narrative review, this study does not aim to provide exhaustive coverage of all published evidence and is inherently subject to selection and interpretive bias. To reduce this risk, we used explicit eligibility criteria, included literature from multiple disciplines, incorporated both empirical studies and policy documents, and sought to include evidence that both supported and challenged a biosocial interpretation. Nonetheless, the review remains susceptible to incomplete retrieval and author interpretation, and these limitations should be considered when interpreting the findings.

Unlike systematic reviews, which are constrained by narrowly defined questions and hierarchies of evidence, narrative reviews are uniquely positioned to synthesize diverse forms of evidence, ranging from empirical data to clinical experience, policy perspectives, and theoretical frameworks, particularly in areas marked by complexity, heterogeneity, and evolving understanding [20]. This flexibility enables the development of broader conceptual linkages and the generation of new hypotheses, which are essential for advancing oral cancer screening that cannot be fully captured through reductionist or purely quantitative approaches.

## 4. Paper Context

- Main findings: The implementation of oral cancer screening faces complex logistical, political, economic, and methodological challenges that are deeply intertwined with social conditions, where those most at risk of oral cancer (low-income, minority populations) paradoxically have the least access to screening services.
- Added knowledge: While previous research focuses primarily on technical aspects of screening and cost-effectiveness, this paper provides the first comprehensive biosocial analysis of oral cancer screening barriers, revealing how social determinants, healthcare provider biases, and political–economic factors collectively influence screening access and outcomes globally.
- Global health impact for policy and action: The paper’s proposed three-pillar framework (multi-domain initiatives, systematic policy modification, and rigorous empirical investigation) provides actionable guidance for policymakers to expand screening beyond traditional dental settings, reform healthcare delivery systems, and prioritize vulnerable populations.

## 5. Conclusions

This review examines oral cancer screening through a biosocial lens to explore how clinical, structural, and policy factors shape access to early detection. The findings suggest that current screening practices are inconsistent, guideline recommendations vary across settings, and populations at highest risk often face the greatest barriers to access. These patterns indicate that oral cancer screening cannot be understood only as a technical or clinical issue but also as one shaped by healthcare organization, evidentiary standards, and broader social conditions. The global burden of oral cancer demands immediate and decisive action to transform our inadequate screening practices. Each year, thousands of preventable deaths occur due to late detection, particularly in underserved communities. A more equitable approach to oral cancer screening may therefore require closer attention to workforce training, service integration, guideline development, and the structural barriers affecting underserved populations. Future research and policy work should continue to evaluate how screening frameworks can be adapted to different health system contexts while minimizing unintended consequences and improving access to early detection.

## Data Availability

No new data were created or analyzed in this study. Data sharing does not apply to this article.

## References

[1] Lingen M.W., Kalmar J.R., Karrison T., Speight P.M. (2008). Critical evaluation of diagnostic aids for the detection of oral cancer. Oral Oncol..

[2] Rivera C. (2015). Essentials of oral cancer. Int. J. Clin. Exp. Pathol..

[3] Gupta N., Gupta R., Acharya A.K., Patthi B., Goud V., Reddy S., Garg A., Singla A. (2017). Changing Trends in oral cancer—A global scenario. Nepal J. Epidemiol..

[4] Varela-Centelles P. (2022). Early Diagnosis and Diagnostic Delay in Oral Cancer. Cancers.

[5] Warnakulasuriya S. (2009). Significant oral cancer risk associated with low socioeconomic status. Evid.-Based Dent..

[6] Brandizzi D., Chuchurru J., Lanfranchi H., Cabrini R. (2005). Analysis of the epidemiological features of oral cancer in the city of Buenos Aires. Acta Odontol. Latinoam..

[7] Natarajan P.M., Swamikannu B., Sivaraman N.M., Stylin A.G.S.Q. (2024). Prevention of Oral Cancer: A Comprehensive Guide. J. Pharm. Bioallied Sci..

[8] National Cancer Institute (2022). The Surveillance Epidemiology and End Results. Cancer Stat Facts: Oral Cavity and Pharynx Cancer.

[9] Semprini J. (2022). Oral cancer screening prevalence in low-income adults before and after the ACA. Oral Oncol..

[10] Howlader N., Noone A.M., Krapcho M., Miller D., Brest A., Yu M., Ruhl J., Tatalovich Z., Mariotto A., Lewis D.R. (2020). SEER Cancer Statistics Review, 1975–2017.

[11] Kavyashree C., Vimala H., J. S. (2024). A systematic review of artificial intelligence techniques for oral cancer detection. Healthc. Anal..

[12] Epstein J.D., Knight T.K., Epstein J.B., Bride M.A., Nichol M.B. (2007). Cost of care for early- and late-stage oral and pharyngeal cancer in the California Medicaid population. Head Neck.

[13] Sineshaw H.M., Ellis M.A., Yabroff K.R., Han X., Jemal A., Day T.A., Graboyes E.M. (2020). Association of Medicaid Expansion Under the Affordable Care Act With Stage at Diagnosis and Time to Treatment Initiation for Patients With Head and Neck Squamous Cell Carcinoma. Arch. Otolaryngol. Neck Surg..

[14] Osazuwa-Peters N., Barnes J.M., Megwalu U., Boakye E.A., Johnston K.J., Gaubatz M.E., Johnson K.J., Panth N., Sethi R.K., Varvares M.A. (2020). State Medicaid expansion status, insurance coverage and stage at diagnosis in head and neck cancer patients. Oral Oncol..

[15] Sankaranarayanan R., Ramadas K., Thara S., Muwonge R., Thomas G., Anju G., Mathew B. (2013). Long term effect of visual screening on oral cancer incidence and mortality in a randomized trial in Kerala, India. Oral Oncol..

[16] D’cRuz A.K., Vaish R. (2021). Risk-based oral cancer screening—Lessons to be learnt. Nat. Rev. Clin. Oncol..

[17] Agarwal P., Agrawal R.R., Jones E.A., Devaiah A.K. (2020). Social Determinants of Health and Oral Cavity Cancer Treatment and Survival: A Competing Risk Analysis. Laryngoscope.

[18] Akinkugbe A.A., Garcia D.T., Brickhouse T.H., Mosavel M. (2020). Lifestyle risk factor related disparities in oral cancer examination in the U.S: A population-based cross-sectional study. BMC Public Health.

[19] Ramos L., Sobrinho A., Ribeiro L., Martins-De-Barros A., Maurício H., Ferreira S., Carvalho M.d.V. (2022). Racial disparity and prognosis in patients with mouth and oropharynx cancer in Brazil. Med. Oral Patol. Oral Y Cirugia Bucal.

[20] Agarwal S., Charlesworth M., Elrakhawy M. (2023). How to write a narrative review. Anaesthesia.

[21] Turner D.P., Winn R.A., Findlay V.J. (2024). Biosocial determinants inform on enduring cancer disparities. Trends Cancer.

[22] Bray F., Laversanne M., Sung H., Ferlay J., Siegel R.L., Soerjomataram I., Jemal A. (2024). Global cancer statistics 2022: GLOBOCAN estimates of incidence and mortality worldwide for 36 cancers in 185 countries. CA A Cancer J. Clin..

[23] World Health Organization (2022). Global Oral Health Status Report: Towards Universal Health Coverage for Oral Health by 2030.

[24] Sofi-Mahmudi A., Masinaei M., Shamsoddin E., Tovani-Palone M.R., Heydari M.-H., Shoaee S., Ghasemi E., Azadnajafabad S., Roshani S., Rezaei N. (2021). Global, regional, and national burden and quality of care index (QCI) of lip and oral cavity cancer: A systematic analysis of the Global Burden of Disease Study 1990–2017. BMC Oral Health.

[25] (2023). GBD 2019 Lip, Oral, and Pharyngeal Cancer Collaborators. The Global, Regional, and National Burden of Adult Lip, Oral, and Pharyngeal Cancer in 204 Countries and Territories. JAMA Oncol..

[26] Mathunny M.M.S., Sivakumar R., Padmakumar S.K. (2024). A comparative analysis of the burden of lip and oral cavity cancers in the Indian subcontinent. J. Oral Maxillofac. Pathol..

[27] Kleinman A. (2010). Four social theories for global health. Lancet.

[28] Bouvard V., Nethan S.T., Singh D., Warnakulasuriya S., Mehrotra R., Chaturvedi A.K., Chen T.H.-H., Ayo-Yusuf O.A., Gupta P.C., Kerr A.R. (2022). IARC Perspective on Oral Cancer Prevention. N. Engl. J. Med..

[29] Johnson N. (2001). Tobacco Use and Oral Cancer: A Global Perspective. J. Dent. Educ..

[30] IARC (2023). Oral cancer prevention. IARC Handbooks of Cancer Prevention.

[31] American Cancer Society Risk Factors for Oral Cavity and Oropharyngeal Cancers 2021. https://www.cancer.org/cancer/oral-cavity-and-oropharyngeal-cancer/causes-risks-prevention/risk-factors.html.

[32] Lee Y.-C.A., Al-Temimi M., Ying J., Muscat J., Olshan A.F., Zevallos J.P., Winn D.M., Li G., Sturgis E.M., Morgenstern H. (2020). Risk Prediction Models for Head and Neck Cancer in the US Population From the INHANCE Consortium. Am. J. Epidemiol..

[33] Marron M., Boffetta P., Zhang Z.-F., Zaridze D., Wünsch-Filho V., Winn D.M., Wei Q., Talamini R., Szeszenia-Dabrowska N., Sturgis E.M. (2009). Cessation of alcohol drinking, tobacco smoking and the reversal of head and neck cancer risk. Leuk. Res..

[34] De Stefani E., Boffetta P., Deneo-Pellegrini H., Ronco A.L., Acosta G., Ferro G., Oreggia F., Leiva J. (2006). The effect of smoking and drinking in oral and pharyngeal cancers: A case-control study in Uruguay. Cancer Lett..

[35] Radoï L., Paget-Bailly S., Cyr D., Papadopoulos A., Guida F., Schmaus A., Cénée S., Menvielle G., Carton M., Lapôtre-Ledoux B. (2013). Tobacco smoking, alcohol drinking and risk of oral cavity cancer by subsite: Results of a French population-based case-control study, the ICARE study. Eur. J. Cancer Prev..

[36] Warnakulasuriya S., Kerr A. (2021). Oral Cancer Screening: Past, Present, and Future. J. Dent. Res..

[37] Warnakulasuriya S., Greenspan J.S. (2020). Epidemiology of oral and oropharyngeal cancers. Textbook of Oral Cancer, Editor.

[38] Gupta A., Sonis S., Uppaluri R., Bergmark R.W., Villa A. (2019). Disparities in Oral Cancer Screening Among Dental Professionals: NHANES 2011–2016. Am. J. Prev. Med..

[39] Coppola N., Mignogna M.D., Rivieccio I., Blasi A., Bizzoca M.E., Sorrentino R., Muzio L.L., Spagnuolo G., Leuci S. (2021). Current Knowledge, Attitudes, and Practice among Health Care Providers in OSCC Awareness: Systematic Review and Meta-Analysis. Int. J. Environ. Res. Public Health.

[40] Applebaum E., Ruhlen T.N., Kronenberg F.R., Hayes C., Peters E.S. (2009). Oral cancer knowledge, attitudes and practices: A survey of dentists and primary care physicians in Massachusetts. J. Am. Dent. Assoc..

[41] Gigliotti J., Madathil S., Makhoul N. (2019). Delays in oral cavity cancer. Int. J. Oral Maxillofac. Surg..

[42] Galeș L.N., Păun M.-A., Anghel R.M., Trifănescu O.G. (2024). Cancer Screening: Present Recommendations, the Development of Multi-Cancer Early Development Tests, and the Prospect of Universal Cancer Screening. Cancers.

[43] Dwojak S., Bhattacharyya N. (2017). Racial disparities in preventable risk factors for head and neck cancer. Laryngoscope.

[44] Massachusetts Health Policy Commission HPC DataPoints, Issue 20: Oral Health Access and Equity in the Commonwealth 2021. https://masshpc.gov/publications/datapoints-series/issue-20-oral-health-access-and-equity-commonwealth.

[45] Moyer V.A. (2014). U.S. Preventive Services Task Force. Screening for Oral Cancer: U.S. Preventive Services Task Force Recommendation Statement. Ann. Intern. Med..

[46] American Cancer Society Can Oral Cavity and Oropharyngeal Cancers Be Found Early? 2021. https://www.cancer.org/cancer/oral-cavity-and-oropharyngeal-cancer/detection-diagnosis-staging/detection.html.

[47] Lingen M.W., Abt E., Agrawal N., Chaturvedi A.K., Cohen E., D’sOuza G., Gurenlian J., Kalmar J.R., Kerr A.R., Lambert P.M. (2017). Evidence-based clinical practice guideline for the evaluation of potentially malignant disorders in the oral cavity: A report of the American Dental Association. J. Am. Dent. Assoc..

[48] Speight P., Warnakulasuriya S., Ogden G. (2010). Early Detection and Prevention of Oral Cancer: A Management Strategy for Dental Practice.

[49] Farmer P., Kleinman A., Kim J., Basilico M. (2013). Reimagining Global Health: An Introduction.

[50] Burns P.B., Rohrich R.J., Chung K.C.M. (2011). The Levels of Evidence and Their Role in Evidence-Based Medicine. Plast. Reconstr. Surg..

[51] Johnson N., Warnakulasuriya S., Gupta P., Dimba E., Chindia M., Otoh E., Sankaranarayanan R., Califano J., Kowalski L. (2011). Global oral health inequalities in incidence and outcomes for oral cancer: Causes and solutions. Adv. Dent. Res..

[52] Fonseca S. (2020). Latin American Social Medicine: The Making of a Thought Style. Ph.D. Thesis.

[53] National Cancer Institute Cancer Disparities 2022. https://www.cancer.gov/about-cancer/understanding/disparities.

[54] Dantas M.N.P., de Souza D.L.B., de Souza A.M.G., Aiquoc K.M., de Souza T.A., Barbosa I.R. (2021). Fatores associados ao acesso precário aos serviços de saúde no Brasil. Rev. Bras. Epidemiol..

[55] CDC Disparities in Oral Health. Division of Oral Health, National Center for Chronic Disease Prevention and Health Promotion 2021. https://www.cdc.gov/oral-health/health-equity/?CDC_AAref_Val=https://www.cdc.gov/oralhealth/oral_health_disparities/index.htm.

[56] Massachusetts Department of Public Health (2021). Oropharyngeal Cancer: Status in Massachusetts.

[57] Mukherjee J.S. (2021). Understanding and Practicing Social Medicine. An Introduction to Global Health Delivery.

[58] Farmer P. (1995). Dr. Paul Farmer: Medicine and liberation theology. America Magazine.

[59] Pierson L., Verguet S. (2023). When should global health actors prioritise more uncertain interventions?. Lancet Glob. Health.

[60] Farmer P.E. (2004). Chapter 5 Health, Healing, and Social Justice Insights from Liberation Theology Making a Preferential Option for the Poor. Pathologies of Power: Health, Human Rights, and the New War on the Poor.

[61] Kasper J., Greene J.A., Farmer P.E., Jones D.S. (2016). All Health Is Global Health, All Medicine Is Social Medicine: Integrating the Social Sciences Into the Preclinical Curriculum. Acad. Med..

[62] Kleinman A. (2019). The Soul of Care: The Moral Education of a Husband and a Doctor.

[63] Stahl S., Meskin L.H., Brown L.J. (2004). The American Dental Association’s oral cancer campaign: The impact on consumers and dentists. J. Am. Dent. Assoc..

[64] Maybury C., Horowitz A.M., Goodman H.S. (2012). Outcomes of oral cancer early detection and prevention statewide model in Maryland. J. Public Health Dent..

